# 3Avoiding the 'twilight zone': recommendations for the transition of services from adolescence to adulthood for young people with ADHD

**DOI:** 10.1186/1471-244X-11-174

**Published:** 2011-11-03

**Authors:** Susan Young, Clodagh M Murphy, David Coghill

**Affiliations:** 1 Department of Forensic and Neurodevelopmental Sciences, King's College London, Institute of Psychiatry, De Crespigny Park, London, SE5 8AF, UK; 2 Centre for Neuroscience, Division of Medical Sciences, College of Medicine, Dentistry & Nursing, Ninewells Hospital & Medical School, University of Dundee, DD1 9SY, UK

## Abstract

Attention deficit hyperactivity disorder (ADHD) is a common childhood disorder that frequently persists into adulthood. However, in the UK, there is a paucity of adult services available for the increasing number of young people with ADHD who are now graduating from child services. Furthermore, there is limited research investigating the transition of young people with ADHD from child to adult services and a lack of guidance on how to achieve this effectively. This paper reviews the difficulties of young people with ADHD and their families who are transitioning between services; we review transition from the child and adult health teams' perspectives and identify barriers to the transition process. We conclude with recommendations on how to develop transition services for young people with ADHD.

## Background

ADHD affects around 3-4% of UK children [1] and has a wide-ranging and detrimental impact on the wellbeing of individuals who may have a range of clinical, neuropsychological and psychosocial problems [2]. Common comorbid problems in childhood include oppositional defiant disorder (40%), anxiety disorder (34%), conduct disorder (14%), tics (11%) and mood disorder (6%) [3,4]. As children develop, many continue to suffer impairment from their symptoms. A meta-analysis of follow-up studies conducted by Faraone and colleagues [5] found that around 15% of cases continue to meet diagnostic criteria for ADHD at 25 years of age, with a further 50% of individuals suffering impairment from residual symptoms of ADHD. Comorbid problems also persist and/or develop afresh, including anxiety, mood problems and substance misuse [6-8]. The presentation of ADHD in adults may be complicated by the chronicity of their ADHD symptoms, and associated difficulties including low self-esteem, interpersonal relationship problems, educational and occupational difficulties, risk taking behaviours, driving accidents, delinquency and offending; even when ADHD has been recognised and treated, outcomes are often somewhat bleak [9,10]. These individuals are further disadvantaged by their cognitive and social deficits, impulsivity and poor attention, and may experience greater difficulty in achieving autonomy than their peers. Thus the transfer between child and adult services occurs at a time of increased vulnerability, when young people with ADHD may require guidance and support from trusted carers, including health care professionals. Data from the Multimodal Treatment of ADHD (MTA) study clearly suggests that well thought through and organized evidence based treatment protocols can improve outcomes for those with ADHD [11,12]. However, as ADHD has not yet been widely embraced by adult mental health services in the UK, many are untreated [13] and there are limited established clinical services offering planned transition to adult teams for young people with ADHD. These service provision limitations, together with the symptoms and complexities of young people with ADHD, make the transition process harder to resolve, and necessitate unique solutions compared with other better accepted mental health disorders.

Within this context we will focus our discussion on the barriers to the transition process, the care gap between child and adult services, current models of transition and conclude with service recommendations.

## Barriers to transition

Whilst the long-term risks associated with persisting ADHD highlight the importance of maintaining treatment and engagement with health services [13], during the crucial period of transition to adulthood the opposite occurs and there is almost complete disengagement from services by age 21 [14]. This is unlikely to reflect spontaneous symptom remission as around two-thirds of ADHD children will continue to suffer impairment of symptoms at age 25 [5]. Although this may to some degree reflect a conscious decision by young people to opt out of treatment it is likely that several other factors may contribute to this decline in service utilisation, including a relative lack of transition services, difficulties for young people in coping with transition and/or feeling let down by services. Whilst some adults may present later on in life with serious mental health problems [9] it is likely that many continue to suffer alone without healthcare. They will, however, often continue to make demands on the other parts of the healthcare system at significant cost to themselves and society (e.g. increased rates of medical admissions/attendance in Accident and Emergency Departments, the criminal justice system, Departments of Employment, Learning and Social Services) [15,16].

The 'TRACK' survey examined policies and practices in Greater London for the transition of care from child and adolescent mental health services (CAMHS) to adult mental health services (AMHS) [17]. They conclude that the complexity of service structures, arbitrary service boundaries, variation in protocols and a possible policy-practice gap all contribute to a discontinuity of mental healthcare for a significant number of young people who experience no or poor transition of care across services. However, inadequate protocols and poor service provision may not be solely responsible for the care gap. Lay and professional misunderstandings and misinformation about ADHD abound, and may contribute to differences that exist between CAMHS and adult services in theoretical and conceptual views of diagnosis, cause and treatment focus [18]. ADHD is not included in mainstream training for many healthcare professionals, including psychology, nursing and medical training. There is a clear need for increased multidisciplinary education about ADHD at both an undergraduate and post-graduate level. Additionally, cultural differences in attitudes and values between child and adult practices may hamper the collaborative arrangements for transferring patients. Importantly, differences in conceptual models of practice may exist, with CAMHS adopting a developmental perspective and AMHS a more medical approach [19].

Service user involvement in service planning and development helps to ensure that this is based on the needs of the young people who use them [19]. There is little research on service user and carer experiences, the outcomes of individuals who fall through care gaps, or about interventions that might improve the process of transition. The small evidence base that is available suggests that the outcome of stopping treatment in adolescence is dependent on several factors: recurrence of symptoms, residual symptoms and ability to re-engage with services, family circumstances, and educational/work circumstances [13]. The patients who reported the most satisfactory outcomes from cessation tended to be those who had planned the process with their clinician. Most psychosocial treatments in childhood are currently indirect interventions (e.g. parent training, classroom interventions) and oftentimes young people presenting to adult services have never been spoken to directly about their symptoms and associated problems. Also, parents who have supported their child in treatment for many years will experience a change in their own role and may suddenly feel unimportant and shut out of the process. The result may be that both parties - parent and child - feel anxious about the future. In turn, and with increasing distress, relationships may become strained and unsupportive. Thus it is important that practitioners are sensitive to the changing dynamic as both parents and children shift not only from one style of service provision to another but in their own family roles.

Adult ADHD has a high familial load; approximately 20% of parents of children with ADHD have ADHD themselves [20]. This may significantly impact on their ability to model organisational skills for their children (e.g. completing healthcare forms, replying to letters from health teams, remembering to take medication) and may contribute to missed appointments. Likewise, familial ADHD may further challenge families who, faced with unclear pathways for transition to adult care, have to navigate through a quagmire of healthcare bureaucracy to find appropriate adult healthcare for their adolescent. Both child and adult teams should be mindful of the impact of possible parental ADHD on the transition process and provide clear structured support to families in transition.

## The care gap between child and adolescent services

In the UK, healthcare for children with ADHD is usually provided by either paediatric services or by CAMHS, depending on local arrangements. There are good examples of joint working in some regions, nevertheless, in most areas the bulk of the service is provided by one professional group, with little movement of patients between the two. Traditionally paediatric services stopped relatively early in adolescence. However, in recent years most paediatric services have agreed to provide care until school leaving age. This may or may not correspond with the agreed age of transition from CAMHS to AMHS. Whilst in the past the bulk of CAMHS services stopped at either 16 years of age or school leaving (whichever was later) there is now a shift in policy towards CAMHS services retaining responsibility for care until 18 years of age. Anecdotal discussions with clinicians across the UK would suggest that whatever the technical cut off age, many paediatric and CAMHS teams continue to see young people well past this age due to perceived difficulties transferring care to adult services. Although this does allow some young patients to access continued care for a limited period, the lack of clarity for both patients and professionals is confusing. The National Institute for Clinical Health Excellence ADHD guidelines (NICE) have made clear recommendations that young people with ADHD are re-assessed at school leaving age using a Care Program Approach to determine if continued treatment is required [21].

There has been increased acknowledgment by some that ADHD often persists into adulthood. However, many adult mental health professionals remain sceptical about the validity of ADHD as a true disorder and in particular as an adult disorder [22,23]. This issue of validity of ADHD in adults was addressed by the NICE ADHD Guideline group who concluded that ADHD is a valid disorder that continues into adulthood and that adults with ADHD should be identified and managed within the UK's National Health System [21]. Three main categories of service provision for adults with ADHD were identified [21]. Firstly the 'transition group' consisting of young adults who were diagnosed and treated for ADHD in childhood and still require treatment. These individuals may be stable on medication and require monitoring; stable on medication but with comorbid problems that require additional drug and/or psychological treatments; or unstable on their current treatment. The second category is adults who were diagnosed in childhood but who are currently untreated. These individuals are often those who have disengaged with childhood services but re-present in adulthood, often following a crisis (e.g. threat of relationship breakdown, occupational problems). This may also include a group who have continued to attend but have chosen to stop treatment. The third category consists of adults who are presenting for the first time for assessment. Their presentation to services frequently appears to be triggered following their child's diagnosis with ADHD and recognition that their own difficulties may be related to ADHD and/or following a history of employment, academic or relationship difficulties that seem at variance with the individual's potential. Thus adult services are required to provide a service not only for young people with ADHD transferring from child and adolescent services but also for those who are presenting for the first time as adults or those who have 'fallen out' of treatment and are re-presenting as adults. Yet, at the moment, clinical experience suggests that many adults with ADHD do not receive services from adult mental health teams who perceive ADHD as falling outside of their remit.

Indeed a commonly encountered problem faced by those referring to AMHS is the accepting team's referral criteria, which typically require the presence of "enduring mental health problems". This seems to be a hybrid of the term 'severe and enduring mental illness', used by adult services, and 'mental health problems', a term used more by CAMHS [24]. If an adult mental health service believes that neurodevelopmental disorders fall outside of this criterion then many individuals with ADHD, and other developmental disorders such as autism and mild to moderate learning disability, are likely to fall through the care net.

In the UK, NICE [21] recommended that transition is completed by age 18 which, if one assumes that 16 would be the youngest age for transition, allows a two year window for this to be achieved. In reality, many child services remain cautious about transferring their patients to an adult mental health service and/or they have difficulty having them accepted by these services. Thus they maximise the existing collaboration with child and family by 'holding on' to their developing adolescents and some continue to treat them into young adulthood. Given the data from the General Practice Research Database [14], it would appear that this practice does not facilitate continued engagement with treatment as the vast majority of young people discontinue treatment by age 21.

## Current models of Transition

There are currently two main models of transition between CAMHS and AMHS in the UK; (1) Using a "transition team" that operates independently from CAMHS and AMHS to bridge the gap, or (2) the use of shared care protocols during which CAMHS and AMHS interlock and facilitate a gradual transfer of care. There is precedent for the independent transition service model as this has been implemented in early intervention in psychosis, albeit with mixed success [25,26]. One disadvantage of this model is the introduction of additional and unnecessary divides within the system. The interlocking model is consistent with the National CAMHS review [27], which concluded that transition should be flexible to the needs of young adults rather than focusing specifically on chronological age. It can therefore be paced against the needs of the individual.

Taylor et al. [28] discussed transition for those with ADHD from a paediatric perspective. They proposed a three tiered model of care for transitioning young people whereby the pathway is determined for each individual based on the level of complexity and need. They suggest that those with good symptom control could be managed by general practitioners (GPs) alone, with facilitated access back to specialist services available if required. The second tier is for young people with more complex needs and involves a shared-care protocol between GPs and specialist nurses. In this model, specialist nurses take a pivotal role as the clinical lead in providing support for young people and their families to facilitate transition. They act as a 'skilled bridge' between GPs and adult mental health services. The third tier is for those with ongoing mental health needs (e.g. comorbidities such as depression, anxiety, Asperger's Syndrome) who require specialist services for assessment and intervention, and who would be managed by specialist care pathways within adult mental health together with the availability of input from student and occupational health services where appropriate. From a case note review of their own caseload, Taylor et al. suggest that 5% of their patients could be discharged rather than referred on, 29% could be referred back to the GP, 29% would require shared care between a specialist nurse and the GP, and 36% would require AMHS (30% general adult, 6% learning disability). By definition those patients that would be suitable for GP-only care are the least complex cases, but it is very likely that most GPs would require some training in ADHD and its management, including the recognition and management of common comorbidities and associated problems. One way to provide such training would be through an initial period of support from specialist nurses; although this will take time to develop as whilst there are many skilled specialist nurses working within child and adolescent ADHD care pathways, there are currently few whose experiences bridge both ADHD and adult mental health problems. Another option would be to develop a cohort of GPs with a special interest in developmental disorders, as occurs for a wide range of physical health problems. One additional concern is that the multiple pathways approach may increase the likelihood that young people (who are often ambivalent about the need for continuing care) fall through the care gap and become lost to follow-up.

The rates of comorbid mental health problems were considerably lower in Taylor et al.'s [28] paediatric clinical sample than would be expected from the literature. Thus the proportion of patients requiring follow up by mental health services may be higher in other clinical populations, and it is possible that CAMHS and paediatric services are seeing different groups. Yet even within these two broad groupings there will be patients with very different profiles with respect to severity of core ADHD symptoms, prevalence of psychiatric and physical comorbidities, associated social and educational problems and treatment. These differences may arise as a consequence of differential referral patterns to different services or differences in the skills, approaches, training or philosophy of different professional groups and regions. It is essential that these issues are taken into account by the planning process for ADHD services in general and for transition services in particular. Where a significant mismatch is identified between the observed pattern of associations and those expected from the literature, the service needs to review whether this arises as a consequence of either pre or post-referral practices, and whether changes to practice should be considered.

## Service recommendations

The NICE guidelines on ADHD [21] were developed by a multi-disciplinary professional group with expertise spanning CAMHS, paediatrics, AMHS, and education services. The guidance emphasises that ADHD is a lifespan condition and, for the first time in the UK, provides Guidelines for the development of transition services for this group as follows:

1. Transfer from CAMHS to adult services if patients continue to have significant symptoms of ADHD or other coexisting conditions that require treatment.

2. Transition should be planned in advance by referring and receiving services.

3. Patients should be reassessed at school leaving age and if treatment is necessary arrangements should be made for a smooth transition to adult services.

4. Timings of transition may vary but should be completed by 18 years.

5. During transition, CAMHS/paediatrics and adult services should consider meeting and full information about adult psychiatric services should be made available to the young person.

6. For young people age 16 or over CPA should be used as an aid to transfer.

7. After transition a comprehensive assessment should be carried out and patients should also be assessed for any coexisting conditions.

8. Trusts should ensure that specialist ADHD teams for children, young people and adults jointly develop age-appropriate training programmes for diagnosis and management of ADHD

This acknowledgement of ADHD as a lifelong condition has naturally led to a need for recommendations about how to best engage young people and achieve a smooth transition between child and adolescent services and adult mental health services, and general guidelines have also been produced, for example by the National Mental Health Development Unit [29].

It is almost certainly the case that there is no single 'ideal' template for ADHD transition services. Different situations will require different solutions. However, we do believe that certain general practice points that cut across different patterns of service delivery should be taken into account when setting up such services. We have therefore extended and further developed the NICE Guidelines for commissioners and providers of healthcare services on the transition of young people from child to adult services. These are summarised as follows:

1. ADHD often continues into adulthood. A significant proportion of young people with ADHD will continue to need support and treatment from health service professionals when they reach adulthood.

2. Transition should be planned in advance by both referring and receiving services.

3. Timings of transition may vary but should ordinarily be completed by 18 years. Transition between teams should be a gradual process, e.g. a minimum period of six months.

4. ADHD services for children and adolescents vary considerably between regions (e.g. CAMHS, paediatrics, availability of shared care). It is essential that commissioners take local resources into account when designing transition service in order that realistic and deliverable provisions can be made within services that are often required to work at high capacity within strict budgets.

5. Clinicians providing services for children, young people and adults should ensure they keep abreast of evidence-based, up-to-date recommendations about the diagnosis and management of ADHD at different developmental stages as part of their continuing professional development.

6. A planned transfer to an appropriate adult service should be made if the young person continues to have significant symptoms of ADHD or other co-existing conditions that require treatment.

7. Appropriate adult services should include primary care, adult community mental health teams and access to specialist adult ADHD services.

8. Clear transition protocols should be developed jointly by commissioners, CAMHS/paediatric services, AMHS and primary care to facilitate transition and ensure standards of care are maintained during the transition period. These protocols should be developed with service users' involvement to ensure they meet the needs of the young people who will use them.

9. These transition protocols should be available to all clinical teams and should include psychoeducational material that provides high quality, comprehensive, impartial and appropriately written information for both young people and their parents/carers. This material should include information about ways that young people can manage their own symptoms and problems, and access advice and support. Information should also be developed in a media format that is readily accessed by young people, e.g. use of phone applications and internet sites.

10. Pre-transition: young people with ADHD should be reassessed at school leaving age by the service managing their care. They should be informed of the outcome of this assessment and transitioned according to need, e.g. to GP services, adult community mental health teams (community, learning disability or forensic as appropriate), specialist adult ADHD teams, or adult physical health teams where required. Both the patient and all adult/GP teams receiving referrals should be jointly informed of the patient's initial transition.

11. During transition: child and adult services should ideally have a joint transition appointment. Full information about adult psychiatric and GP services should be made available to the young person and their family. Full information about the young person's paediatric/CAMHS care should be available to the adult teams, including a detailed clinical transition report.

12. CAMHS practitioners and paediatricians should foster engagement with AMHS through open discussion and psychoeducation about ADHD, the benefit of evidenced based psychological and pharmacological treatment where appropriate, and the risks of disengagement. It is important to address concerns about stigma associated with referral to AMHS.

13. Joint meetings between child and adult services must ensure the needs of the young person will be appropriately met. This may involve further discussion and collaboration with educational and/or occupational agencies.

14. For young people age 16 or over in CAMHS, care in the UK 'Care Programme Arrangements' (CPA) should be used as an aid to transfer. CPA's are not available in paediatric practice and so a planned assessment of need with the young person and their parent and a clearly documented plan of action is recommended.

15. Parents and carers need to be prepared and facilitated to aid their children's gradually increasing independence and autonomy with their ADHD and its' treatment. Referring child and receiving adult/GP teams should be mindful of possible parental ADHD and support and manage this appropriately.

16. Post transition: a comprehensive assessment should be carried out by the receiving service. Patients should be re-assessed for any coexisting conditions and referred for assessment/treatment/support of associated difficulties, including co-morbid mental health/learning/educational/employment support.

17. Shared care arrangements between primary and secondary care services for the prescription and monitoring of ADHD medications should be continued into adulthood.

18. Direct psychological treatment should be considered (individual and/or group CBT) to support young people during key transitional stages. This should have a skills development focus and target a range of areas including social skills, interpersonal relationship problems (with peers and family), problem solving, self-control, listening skills and dealing with and expressing feelings. Active learning strategies should be used (e.g. see [30-32]).

19. Direct psychological treatment should be considered (individual and/or group CBT) to support young people who are experiencing symptom remission and/or stopping medication.

In developing this guidance, we have drawn on a review of the literature, the NICE guidelines, our clinical experience, and expert opinion. The guidance includes the need to involve service-users' feedback in the development of transition protocols and psychoeducational materials to include the information on self-management of symptoms and problems. Although this guidance should not be seen as prescriptive, we hope it can facilitate the planning process by helping to organize thinking and guide discussions among clinicians and commissioners.

Historically, the role of GPs in managing ADHD in children and adolescents has been restricted to shared care of prescribing with specialists in secondary care; the latter monitoring continuing care whilst GPs write the prescriptions. Indeed the Summary of Product Characteristics for the licensed ADHD medications all indicate the need for specialists to oversee and monitor the use of these medications in individual patients. However, transition patients will have often received many years of specialist care by CAMHS or paediatric services and the GP will have access to significant documentation of this care. Likewise, many GPs will already have been prescribing for this group, with specialist monitoring provided by paediatric/CAMHS teams. Thus it seems acceptable for GPs to manage a proportion of transitioning patients whose ADHD is stable on treatment, much as they manage cases of anxiety or depression. This again highlights the importance of primary care staff being provided with relevant training and adequate support, as well as the need to facilitate a quick and easy route back into specialist services if necessary. Likewise, specialist nurses can make a very important and helpful contribution to the management of adults with ADHD, as long as they are well trained in both ADHD and adult mental health problems and are given adequate support. However, it will still be necessary for a considerable proportion of patients to have their care managed by general AMHS, with a proportion of patients also referred to specialist adult ADHD services as required. Experience from managing children and adolescents with ADHD suggests that one potential model of care for this group would comprise a single care pathway, with agreed protocols for assessing and monitoring core ADHD symptoms, comorbid mental health, physical problems, common associated difficulties (e.g. relationship problems and occupational/academic problems), overall impairment, and managing both pharmacological and non-pharmacological treatments. Within this care pathway there would be different levels of care (e.g. GP only, GP + specialist nurse, AMHS, specialist adult ADHD services) with agreed protocols to assist decisions about who is managed at each level *and *how and when patients should move between levels with as little disruption to care as possible. Transition from child and adolescent services to this pathway should also be clearly described with the possibility of transition occurring at different ages/stages and in different ways as required.

## Conclusions

There is a care gap in service provision for many young people who continue to suffer pervasive and impairing ADHD symptoms and who remain vulnerable to psychosocial adversity. These young people often fall into a 'twilight zone' in their adolescent years. This is particularly unfortunate as this is a time when they are required to make important decisions about their future and strive to develop a personal and social identity, whilst at the same time experiencing considerable emotional turmoil and change. It is at this time that they are most likely to need the support of appropriate health care services [33]. However, this is not being provided for systemic reasons. First, many child services lack cohesion, transition mechanisms are poorly thought out, the needs of the individual and their carers are often neither acknowledged nor adequately addressed, and last but not least there are limited adult services and/or ways to access them. Policies and protocols for the transition of healthcare at such a sensitive time do exist. However, these are often rather general prescriptions that lack specific guidance for implementation at ground level. It is essential that these policies are reviewed and operationalized so that they can be effectively translated into practice. Best practice may be for local services to commission and implement a single, simple, and clear transition pathway that, regardless of whether the young person comes from a paediatric or CAMHS team, provides age-appropriate assessment, triage and transition as required to adult/GP services.

ADHD is a life-long condition and current adult provision is poor. Simply bridging the transition gap will not address the fundamental problem of who should be responsible for the care of patients with adult ADHD. Since the NICE Guidelines [21] raised this need, many AMHS have started to take more interest in the assessment and treatment of ADHD adults, yet service provision across the UK remains patchy in real terms. The proposed GP-AMHS shared protocol merits development. More positively, training in the diagnosis and treatment of ADHD has been endorsed by the Royal College of Psychiatry and is being regularly delivered across the UK by the United Kingdom Adult ADHD Network (UKAAN). This needs to be extended to other mental health practitioners. We acknowledge that the development of a gold standard transition service would require considerable negotiation, planning, support and finance, and that some commissioners and clinicians may have reservations about committing to additional investments in healthcare. However, set against the considerable costs to the individual, family and society that are associated with untreated ADHD, there appear to be clear clinical, ethical and financial arguments that suggest that short-term investment in transition would realize long-term gains.

## List of Abbreviations

**ADHD: **Attention Deficit Hyperactivity Disorder; **AMHS: **Adult Mental Health Services; **ASD: **Autism Spectrum Disorder; **CAMHS: **Child and Adolescent Mental Health Services; **GP: **General Practitioner; **GPRD: **General Practice Research Database; **MTA: **Multimodal Treatment of ADHD; **NHS: **National Health Service; **NICE: **National Institute for Health and Clinical Excellence; **TRACK: **Transitions of care from child and adolescent mental health services to adult mental health services.

## Competing interests

Susan Young has been a consultant for Janssen-Cilag, Eli-Lilly and Shire. She has given educational talks at meetings sponsored by Janssen-Cilag, Shire, Novatis, Eli-Lilly and Flynn-Pharma and has received research grants from National Institute of Health Research, Janssen-Cilag, Eli-Lilly and Shire. She is co-author of 'R&R2 for Youths and Adults with ADHD'. She was a member of the NICE Guideline Development Group for ADHD and is Vice President of UKAAN.

David Coghill has been on advisory boards and/or provided consultancy for Shire, Janssen Cilag, Shering-Plough, Pfizer, Lilly, UCB and Flynn Pharma. He has given educational talks at meetings sponsored by Shire, Janssen Cilag, Medice, Lilly, UCB and Flynn Pharma. He has received research grants from the European Union, Department of Health, National Institute of Health Research, Economic and Social Research Council, Lilly and Shire. He is a member of the UKAAN board.

Clodagh Murphy has no competing interests.

## Authors' information

More information about ADHD, educational forums and training programmes can be found on the UK Adult ADHD Network website (http://www.UKAAN.org).

## Authors' contributions

SY completed the first draft. SY, DC and CM made revisions and edits to subsequent drafts. All authors read and approved the final manuscript.

## Pre-publication history

The pre-publication history for this paper can be accessed here:

http://www.biomedcentral.com/1471-244X/11/174/prepub

## References

[B1] Ford T Goodman R Meltzer H The British Child and Adolescent Mental Health Survey 1999: the prevalence of DSM-IV disorders J Am Acad Child Psy 2003 42 1203 1211 10.1097/00004583-200310000-00011 14560170

[B2] Young S Gudjonsson G Growing out of attention-deficit/hyperactivity disorder: The relationship between functioning and symptoms J Atten Disord 2008 12 162 169 17494827 10.1177/1087054707299598

[B3] MTA Cooperative Group Moderators and Mediators of Treatment Response for Children With Attention-Deficit/Hyperactivity Disorder: The Multimodal Treatment Study of Children With Attention-Deficit/Hyperactivity Disorder Archive Gen Psychiatry 1999 56 1088 96 10.1001/archpsyc.56.12.1088 10591284

[B4] Simonff E Pickles A Charman T Chandler S Loucas T Baird G Psychiatric disorders in children with autism spectrum disorders: Prevalence, Comorbidity, and associated factors in a population-derived sample J Am Acad Child Adolesc 2008 47 921 929 10.1097/CHI.0b013e318179964f 18645422

[B5] Faraone S Biederman J Mick E The age-dependent decline of attention deficit hyperactivity disorder: A meta-analysis of follow-up studies Psychol Med 2006 36 159 165 16420712 10.1017/S003329170500471X

[B6] Barkley RA Fischer M Edelbrock CS Smallish L The Adolescent Outcome of Hyperactive Children Diagnosed by Research Criteria: An 8-Year Prospective Follow-up Study Journal of the American Academy of Child & Adolescent Psychiatry 1990 29 546 587 10.1097/00004583-199007000-00007 2387789

[B7] Biederman J Petty CR Monuteaux MC Fried R Byrne D Mirto T Spencer T Wilens TE Faraone SV Adult Psychiatric Outcomes of Girls With Attention Deficit Hyperactivity Disorder: 11-Year Follow-Up in a Longitudinal Case-Control Study American Journal of Psychiatry 2010 167 409 417 10.1176/appi.ajp.2009.09050736 20080984

[B8] Taylor E Chadwick O Hepinstall E Danckaerts M Hyperactivity and Conduct Problems as Risk Factors for Adolescent Development Journal of the American Academy of Child & Adolescent Psychiatry 1996 35 1213 1226 10.1097/00004583-199609000-00019 8824065

[B9] Dalsgaard S Mortensen PB Frydenberg M Thomsen PH Conduct problems, gender and adult psychiatric outcome of children with attention-deficit hyperactivity disorder British Journal of Psychiatry 2002 181 416 421 10.1192/bjp.181.5.416 12411268

[B10] Langley K Fowler T Ford T Thapar A van den Bree M Harold G Owen M O'Donovan M Thapar A Adolescent clinical outcomes for young people with attention-deficit hyperactivity disorder The British Journal of Psychiatry 2010 196 235 240 10.1192/bjp.bp.109.066274 20194547

[B11] Swanson J Arnold LE Kraemer H Hechtman L Molina B Hinshaw S Vitiello B Jensen P Steinhoff K Lerner M Greenhill L Abikoff H Wells K Epstein J Elliott G Newcorn J Hoza B Wigal T Evidence, interpretation, and qualification from multiple reports of long-term outcomes in the Multimodal Treatment study of Children With ADHD (MTA): part I: executive summary J Atten Disord 2008 12 4 14 10.1177/1087054708319345 18573923

[B12] Swanson J Arnold LE Kraemer H Hechtman L Molina B Hinshaw S Vitiello B Jensen P Steinhoff K Lerner M Greenhill L Abikoff H Wells K Epstein J Elliott G Newcorn J Hoza B Wigal T Evidence, interpretation, and qualification from multiple reports of long-term outcomes in the Multimodal Treatment Study of children with ADHD (MTA): Part II: supporting details J Atten Disord 2008 12 15 43 10.1177/1087054708319525 18573924

[B13] Wong IC Asherson P Bilbow A Clifford S Coghill D DeSoysa R Taylor E Cessation of attention deficit hyperactivity disorder drugs in the young (CADDY)-a pharmacoepidemiological and qualitative study Health technology assessment (Winchester, England) 2009 13 50 1 120 iii-iv, ix-xi, 10.3310/hta13490 19883527

[B14] McCarthy S Asherson P Coghill D Hollis C Murray M Potts L Sayal K de Soysa R Taylor E Williams T Wong ICK Attention-deficit hyperactivity disorder: treatment discontinuation in adolescents and young adults The British Journal of Psychiatry 2009 194 273 277 10.1192/bjp.bp.107.045245 19252159

[B15] Birnbaum HG Kessler RC Lowe SW Secnik K Greenberg PE Leong SA Swensen AR Costs of attention deficit-hyperactivity disorder (ADHD) in the US: excess costs of persons with ADHD and their family members in 2000 Curr Med Res Opin 2005 21 195 206 10.1185/030079904X20303 15801990

[B16] Leibson CL Long KH Economic implications of attention-deficit hyperactivity disorder for healthcare systems Pharmacoeconomics 2003 21 1239 1262 10.2165/00019053-200321170-00002 14986737

[B17] Singh SP Paul M Ford T Kramer T Weaver T Transitions of care from Child and Adolescent Mental Health Services to Adult Mental Health Services (TRACK Study): a study of protocols in Greater London BMC Health Serv Res 2008 8 135 10.1186/1472-6963-8-135 18573214 PMC2442433

[B18] Singh SP Transition of care from child to adult mental health services: the great divide Current Opinion in Psychiatry 2009 22 386 390 10.1097/YCO.0b013e32832c9221 19417667

[B19] Munoz-Solomando A Townley M Williams R Improving transitions for young people who move from child and adolescent mental health services to mental health services for adults: lessons from research and young people's and practitioners' experiences Current Opinion in Psychiatry 2010 23 311 317 10.1097/YCO.0b013e32833a51e2 20520550

[B20] Faraone SV Biederman J Feighner JA Monuteaux MC Assessing symptoms of attention deficit hyperactivity disorder in children and adults: which is more valid? J Consult Clin Psychol 2000 68 830 842 11068969

[B21] National Institute for Health and Clinical Excellence Attention deficit hyperactivity disorder: diagnosis and management of ADHD in children, young people and adults Clinical guideline 72 2008 London

[B22] Asherson P Adamou M Bolea B Muller U Dunn-Morua S Pitts M Thome J Young S Is ADHD a valid diagnosis in adults? Yes British Medical Journal 2010 340 549 10.1136/bmj.c549 20348184

[B23] Moncrieff J Timimi S Is ADHD a valid diagnosis in adults? No British Medical Journal 2010 340 547 10.1136/bmj.c547 20348183

[B24] Singh SP Paul M Islam Z Weaver T Kramer T McLaren S Belling R Ford T White S Hovish K Harley K Transition from CAMHS to Adult Mental Health Services (TRACK): A Study of Service Organisation, Policies, Process and User and Carer Perspectives Report for the National Institute for Health Research Service Delivery and Organisation Programme: London 2010

[B25] McCrone P Knapp M Economic evaluation of early intervention services British Journal of Psychiatry 2009 191 19 22 10.1192/bjp.191.51.s19 18055933

[B26] Turner MA Boden JM Smith-Hamel C Mulder RT Outcomes for 236 patients from a 2-year early intervention in psychosis service Acta Psychiatrica Scandinavica 2009 120 129 137 10.1111/j.1600-0447.2009.01386.x 19392808

[B27] Department for Children Schools and Families and Department of Health National CAMHS Review: Children and young people in mind: the final report of the National CAMHS Review 2008 London

[B28] Taylor N Fauset A Harpin V Young adults with ADHD: an analysis of their service needs on transfer to adult services Arch Dis Child 2010 95 513 517 10.1136/adc.2009.164384 20530525

[B29] National Mental Health Development Unit Planning mental health services for young adults - improving transition A resource for health and social care commissioners 2011 Raffertys

[B30] Young SJ Bramham J ADHD in Adults: A Psychological Guide to Practice 2007 Chichester: John Wiley & Sons

[B31] Young SJ Ross RR R&R2 for ADHD youths and adults. A prosocial competence training program 2007 Ottawa: Cognitive Centre of Canada http://www.cognitivecentre.ca22125790

[B32] Bramham J Young S Bickerdike A Spain D MacCartan D Xenitidis K Evaluation of group cognitive behavioural therapy for adults with ADHD J Atten Disord 2009 12 434 441 10.1177/1087054708314596 18310557

[B33] Young S Amarasinghe JA Practitioner Review: Non-pharmacological treatments for ADHD: A lifespan approach J Child Psychol Psychiatry 2010 51 116 133 10.1111/j.1469-7610.2009.02191.x 19891745

